# Editorial Notes

**Published:** 1921-09

**Authors:** 


### Editorial Notes.

---

**University Appeal Fund.**

THE University of Bristol is organising a great appeal for public support, and urging the claims of higher education with unremitting energy. The value of the University to the life and industries of the neighbourhood has been fully demonstrated since its foundation in 1909.

Cromwell's chaplain, Dr. Dell, saw far ahead when he wrote that it would be "advantageous to the good of all people, to have universities or colleges, one at least in every great town or city in the nation, as in London, York, Bristol, Exeter, Norwich and the like." Now, after the lapse of nearly three hundred years, we begin to realise how true were the words of the old Commonwealth preacher. In support of the appeal the various departments of the University have furnished records of their activities in the past and set out their aims, ambitions, and hopes for the future.

Each department has solid claims on the public for financial aid, not as charity, but as a public investment of money which will yield returns to the public in ways that the shrewdest contributor can scarcely foresee.

The claims of the **Medical Department** are no less urgent than the others. It will serve better perhaps than promises to enlist the interest of the subscribing public in this department of the University if we allude to a few of the medical achievements in Bristol which are now matters of history.

Foremost among the names of Bristol men who have added lustre to the annals of medicine stands John Free,

Rector of St. Michael's on the hill, who, attracted by the revival of learning in Italy, was the first Englishman to study in that country, and eventually settled as a teacher of medicine in the University of Ferrara, dying in Italy in 1465, just after his appointment to be Bishop of Bath.

At the end of the seventeenth century Thomas Dover, the inventor of Dover's Powders and the discoverer of Alexander Selkirk, was practising in Bristol. He offered his services to the newly-formed "Corporation of the Poor" in this city during an epidemic of spotted fever, and was the first physician appointed to St. Peter's Hospital. Although Dover was not a universal favourite in his profession, he could retain the friendship of such men as Defoe and Sydenham.

In 1798 Dr. Thomas Beddoes founded his "Pneumatic Institute" in Dowry Square, where, with Humphry Davy as his assistant, he made extensive experiments with nitrous oxide (Laughing Gas) and other newly-discovered gases. About the same time James Cowles Prichard came to practise in Bristol. In addition to being a pioneer worker in Ethnology and Anthropology, he was a prime mover in founding the Bristol Medical School. Surgery at this time had notable representatives in Bristol, Thomas Baynton being the most original observer of them all. He invented the treatment of spinal tuberculosis by fixation and rest.

Edward Long Fox (1761-1835) was one of the earliest reformers in the treatment of the insane and built the private asylum at Brislington. His grandson, Edward Long Fox (1832-1902), like his forefather, was a noted physician at the Royal Infirmary, and the author of standard works on Neurology in his day. With such men as Percival and Jowett, Albert and Lewis Fry, Henry Acland, Hobhouse and others, Long Fox helped to kindle the fire of enthusiasm that led to the foundation of University College, from which our Bristol University developed.

William Budd won lasting fame by discovering how typhoid fever was spread. His researches into other infectious diseases, including phthisis, were scarcely less notable so that at his death Professor Tyndall wrote, "There was no physician in England during his lifetime who showed anything like his penetration in the interpretation of zymotic diseases."

Many of us recall memories of that illustrious physician Symonds, and John Beddoe, another of Bristol's physicians, famous as an ethnologist far and wide.

More recently in our own generation Greig Smith gained a world-wide reputation as a founder of modern abdominal surgery. Nor has our city been without its famous medical women. Dr. Elizabeth Blackwell was born in Bristol, although it was in America that she studied and became the first woman to obtain a medical qualification in the United States.

These are some of the traditions which inspire medical study in Bristol. These were our forbears, these were our founders. They were founders of more than a mere school of medicine. History will relate how from the foundations they had laid a college arose for the encouragement of general learning which in due course became the University. Needless is it to name colleagues fortunately with us to-day whose work so well maintains the high standing and reputation of the Bristol School of Medicine.

The needs of the University and of the Medical Department are pressing. The time has passed when medicine can be taught by oral precept alone. Teachers require more than experience of disease and the gift of embodying their knowledge in lecture form. Scientific apparatus and equipment are essential for modern instruction in medicine. Without this enthusiasm and energy are of little avail. The provision and upkeep of equipment is expensive. A sum of £100,000

for the Medical Department will barely suffice to enable Bristol to keep the place it has fairly won among the medical schools of the world. On behalf of the University as a whole and of the medical work in particular we appeal to men and women of public spirit and wealth for generous funds to enable " the swift runners who hand over the lamp of life " to transmit from one generation to another the fire kindled in the childhood of the world, at the Promethean altar of science.

**University of Bristol. Postgraduate Studies.**

The work of the Medical Postgraduate Committee is steadily extending, and courses of medical and surgical demonstrations are being held at Trowbridge, Swindon and Dorchester. Enquiries are being made from other districts, and there is every prospect of a wide extension of postgraduate work throughout the West of England.

The Director has received promises of demonstrations from many members of the teaching staff of the University, and synopses of these lectures are now printed and can be forwarded to prospective centres from which inquiries are received. Medical men forming provincial classes will thus have a wide range of choice of subject.

The lectures are generally in abeyance during December and January, but during next spring not only will these classes be resumed in Bristol and in the country, but it is hoped that a week may be devoted in Bristol to postgraduate study in one or other of the special departments.

The Clinical Assistantships available for one month, or thirty attendances convenient to the practitioner, have attracted a number of practitioners, and demonstrated the wide facilities offered by the University and the clinical institutions.

**Royal Infirmary.** At the half-yearly Board of Governors held on Oct. 13th the announcement of the resignation of the President, Mr. H. H. Wills, was received with the greatest regret. Ill-health has compelled Mr. Wills to take this course. During his tenure of office his interest and energy on behalf of the institution have been invaluable, whilst his generous gift of £100,000 in the early part of this year has made a substantial difference to the financial difficulties with which the Infirmary, like all other voluntary hospitals, was faced.

The Almoner's Report presented at the same meeting contains a remark which arrests our attention. The Almoner states that of all the patients admitted to the Institution only one in five is able to pay the very modest contribution for maintenance which is asked for. From this observation, based on most careful enquiry by an experienced officer well qualified to judge, but one conclusion can be drawn, viz. that the payments by patients will not materially reduce the expenses of the Infirmary so long as it continues to provide for the wants of the really necessitous persons for whom the charity was founded and for whom it has hitherto been maintained. The Governors are now confronted with the serious question whether sufficient voluntary contributions will be forthcoming from the more affluent members of the community, or whether they will be compelled to close their beds to the poor and divert the institution from its original purpose, admitting principally or solely those who can pay for admission. It would be intolerable if "ability to pay" should be the passport into our voluntary hospitals. The public, we venture to think, is unaware of the amount of distress in our midst. There is an impression abroad that wages are high and that all classes enjoy a fair measure of prosperity so that the claims of charity are less pressing. The report shows how erroneous this impression is. Funds are urgently needed, and no scheme for payment by patients is going to solve the difficulty of those who through ill-health have fallen into poverty. Unless voluntary contributions are more freely forthcoming the hospitals of our city will not be able to continue their mission for those whose needs are greatest. Hard times are at hand, when it becomes the more incumbent on us to show unwearied mercifulness touching the care of the poor. We must each and all unlock the treasury of our charity to its fullest extent, so that after the example of generations past we may discharge our just debts of benevolence.

**Phyllis Siepmann Memorial Prize.**

Mr. Henry Arthur Siepmann has founded at the Bristol Royal Infirmary an annual prize of the value of £50 to perpetuate the memory of his sister, Miss Phyllis Siepmann, who died from septicæmia contracted whilst discharging her duties as a surgical dresser at the Infirmary. The prize will be awarded annually to that student who in the third year of attendance at the Royal Infirmary shall do best in an examination in medicine and surgery with special reference to diseases of children. By the request of the women students now attending the Infirmary the prize is open to male and female students alike. Phyllis Siepmann was a student endowed with no mean abilities, and possessed a singular charm of disposition. It is well that we should remember not only "famous men . . . leaders of the people by their counsel . . . rich men furnished with ability," but also pay homage to the promise of youth—

> "With its allusions, aspirations, dreams :
> Book with beginnings, story without end."

**Frenchay Park Sanatorium.**

The wise and witty remarks of Sir William Treloar at the opening ceremony of the Frenchay Park Sanatorium on October 5th were encouraging not only as to the overcoming of initial difficulties but in regard to the measure of success which may be looked for. The Alton and Hayling Island Homes for crippled children, of which he is the Founder and President, boast a record of 90 per cent. of successfully treated cases. These are figures worthy of emulation. When the economic situation permits the completion at Frenchay of open-air wards for 100 children, and when the linking up with Alton as contemplated in the full scheme, is effected, Bristol will be on the fair road of friendly rivalry.

The position of this Sanatorium in the general scheme for dealing with Tuberculosis is complementary to the adult sanatorium, for, as emphasised by the Medical Officer of Health, no scheme for controlling tuberculosis can be effective which deals only with its manifestations at one period of life. Among civilised populations infection by the tubercle bacillus is practically universal at an early age, and if guided to a favourable issue and recovery, affords some security against breakdown in after-life. The function of such a sanatorium for children is therefore more strictly preventive than an adult sanatorium, which remains nevertheless necessary for the care and ease of those whose resistance has given way. In emphasising the imperative need for research as an adjunct and integral part of sanatorium treatment Sir William touched upon a vital point. The city of Bristol requires a Municipal Research Laboratory, where the problems of tuberculosis and other imperfectly understood diseases may receive adequate investigation.

**New Building of the Bristol Homœopathic Hospital.**

THE foundation stone of the new hospital was laid by H. R. H. The Prince of Wales on June 10th. Through the generosity of the President, Mr. Melville Wills, Cotham House, standing in its spacious and beautifully-laid-out grounds of several acres, has been adapted and equipped as a temporary Hospital accommodating twenty-five patients. But when the new buildings are completed the existing house will be devoted to administration and the accommodation of the nursing staff, and 52 beds for patients will be provided in the new Hospital. The cost of providing the house and grounds and of the new building is a gift from the President in memory of his son, the late Capt. Bruce Melville Wills, who was killed in a brave attempt to bring in a wounded comrade early in the late war. It is intended that this new Hospital shall be as up to date and as perfectly equipped as possible, and we earnestly hope that the work carried on will be of much benefit to the patients and prove a worthy memorial of the gallant Captain Wills's self-sacrifice and devotion to his country's service.

